# A Case of Cat Scratch Disease with Neuroretinitis in a 16-Month-Old Boy

**DOI:** 10.1155/2022/2841683

**Published:** 2022-10-13

**Authors:** Daisuke Nakata, Sayaka Kakehi, Hiroshi Okada, Atsuhiro Tanikawa, Yoshiaki Shimada, Masayuki Horiguchi, Yasuki Ito

**Affiliations:** ^1^Department of Ophthalmology, Toyokawa City Hospital, Toyokawa, Aichi, Japan; ^2^Department of Ophthalmology, Fujita Health University, School of Medicine, Toyoake, Aichi, Japan

## Abstract

**Purpose:**

We report a case of neuroretinitis associated with cat scratch disease (CSD) in young children.

**Method:**

Case report.

**Results:**

A 16-month-old boy was admitted for a detailed examination and treatment of a fever of unknown origin. Blood tests revealed no significant findings other than a white blood cell count of 16,100/mm^3^ and C-reactive protein level of 9.89 mg/dL. Computed tomography revealed no relevant findings to determine the causative disease. Antibiotic therapy with cefotaxime was initiated; however, the fever did not resolve. The patient was referred to our department for further examination to detect the cause of the fever. Fundoscopy revealed neuroretinitis in the right eye. His mother reported a history of breeding cats. Cat scratch disease (CSD) was suspected based on the clinical course and fundus findings. Cefotaxime was discontinued, and azithromycin, rifampicin, and prednisolone were administered, following which the fever disappeared and fundus findings improved. Immunoglobulin G (IgG) and IgM antibodies against *Bartonella henselae* was positive, leading to a definitive diagnosis of CSD.

**Conclusion:**

Infants cannot complain of decreased visual acuity; therefore, these findings may be overlooked unless a fundus examination is performed. As in this case, the early detection of neuroretinitis by an ophthalmologist may help in the diagnosis of CSD. It is extremely difficult to capture a photograph of the fundus of an infant, and recording with a smartphone is relatively simple and useful for monitoring continuous changes. *Summary*. We describe a case of neuroretinitis associated with cat scratch disease (CSD) that was diagnosed on the basis of fundus findings. The findings suggest the importance of an aggressive ophthalmologic examination when CSD is suspected in young children who are unable to describe their symptoms.

## 1. Introduction

Cat scratch disease (CSD) is a zoonotic disease caused by *Bartonella henselae*, which is transmitted through cat scratches and cat fleas. It is characterized by symptoms such as fever, local lymphadenitis, and redness of the injured area. In recent years, atypical cases with central nervous system and ocular symptoms have been reported [1–10]; the frequency of the complications of neuroretinitis is approximately 1%–2% [1, 2].

It is relatively difficult to photograph the fundus in infants during examination, and it requires expensive equipment. In recent years, the usefulness of anterior segment and fundus photography using smartphones has been reported, which facilitates convenient recording of the ophthalmologic findings [11, 12].

We present a case of neuroretinitis associated with CSD that was diagnosed through the examination of the fundus. Moreover, we observed the progress of neuroretinitis by photographing the fundus using a smartphone.

## 2. Case Presentation

A 16-month-old boy was admitted to our department for the assessment of a fever of unknown origin. The patient's medical history was insignificant. In early October 2021, the patient visited a local doctor with a complaint of a fever of 39°C and was prescribed acetaminophen (100 mg/day). Since the fever persisted, he was admitted to the Department of Pediatrics at Toyokawa City Hospital on October 11, 2021 for a detailed examination and treatment of the fever.

Physical examination on admission revealed a body temperature of 38.2°C and no obvious lymphadenopathy or rash. Blood tests revealed no significant findings other than a white blood cell count of 16,100/mm^3^ and C-reactive protein level of 9.89 mg/dL, indicating an increased inflammatory response. In addition, computed tomography (CT) from the head to the pelvis revealed no lymphadenopathy, liver or intra-abdominal abscess, or any findings that could identify the cause of the fever.

Antibiotic therapy with cefotaxime (100 mg/kg/day) was initiated, but the fever persisted. Echocardiography revealed no evidence of infective endocarditis or Kawasaki disease, and an otolaryngology examination ruled out otitis media and sinusitis. The patient was referred to our department on October 13, 2021 for the assessment of the fever.

**Figure 1:**
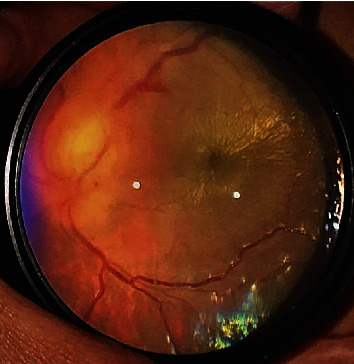
Right eye at the first visit of the examination: papilledema and macular star exudates imaged using a smartphone.

No distinct findings were noted in the anterior segment of the eye. Redness and swelling of the optic disc, serous retinal detachment from the same site to the macula, and macular star exudates were observed in the fundus of the right eye (shown in Figure 1). No abnormalities were observed in the left eye.

The next day, no intracranial lesions were observed under magnetic resonance imaging of the head; the fundus was photographed using a smartphone (iPhone8™, Apple Inc., CA, USA) and a 20-D lens, and the fundus findings were recorded. The shooting method employed was as previously reported [11, 12], and the fundus photograph was captured in the video mode of the smartphone camera application and saved as a still image. The fundus examination revealed neuroretinitis. Upon subsequent enquiry, his mother reported a history of breeding cats and his sister had a history of hospitalization for lymphadenitis. Based on the clinical course and fundus findings, neuroretinitis associated with CSD was suspected, and a *Bartonella* antibody test was performed. Treatment with cefotaxime was discontinued, and azithromycin (10 mg/kg/day), rifampicin (20 mg/kg/day), and prednisolone (1 mg/kg/day) were initiated.

**Figure 2:**
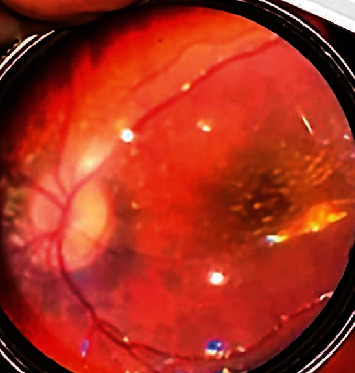
Right eye at the 2-month follow-up after discharge, imaged using a smartphone.

The fever promptly subsided, the fundus findings improved, and the patient was discharged on October 19, 2021. Later, upon testing, the immunoglobulin G (IgG) and IgM antibodies against *Bartonella henselae* were 512 (less than the standard 64 times positive) and 80 (less than the standard 20 times positive), respectively, resulting in a definitive diagnosis of CSD. Antibiotics were administered along with steroids at a tapering dose, and the papilledema of the optic disc and retinal detachment disappeared. No recurrence of optic disc swelling or retinal detachment and no formation of hard exudates in the macula (shown in Figure 2) were observed in the 2-month follow-up. Since then, the patient has been followed-up at an outpatient clinic.

## 3. Discussion

We encountered a case of neuroretinitis associated with CSD, which was not accompanied by local lymphadenopathy and whose fundus findings triggered the identification of the etiology of the patient's fever of unknown origin. Although ocular symptoms are relatively rare in CSD, 5% of the ocular lesions are predominantly conjunctivitis-based anterior ocular inflammation (Parinaud's syndrome), and 1% occur following neuroretinitis or chorioretinitis, which reportedly result in eye inflammation [1–4]. In an epidemiological study of fever of unknown origin in children by Kasai et al. [5], infectious diseases accounted for 23% of all cases, with CSD being the most common. It is important to consider CSD when fever of unknown origin occurs.

Neuroretinitis develops with papilledema and macular edema, and macular star exudates are observed over time. It is caused by various infectious diseases, including ocular toxoplasmosis, tuberculosis, syphilis, and various viral infections (herpes simplex virus, cytomegalovirus, and Epstein-Barr virus), in addition to CSD. Noninfectious diseases include hypertensive retinopathy, sarcoidosis, and Behçet's disease.

In this case, tuberculosis and various viral infections were ruled out by the appropriate blood tests. Sarcoidosis was excluded because chest CT revealed no hilar lymphadenopathy, and Behçet's disease had no extraocular symptoms. At the time of admission to the pediatric department, we did not ask for a history of cat ownership, and a fundus examination revealed optic neuroretinitis, which was additionally considered a differential for CSD. The patient's mother had a history of breeding cats, which was mentioned in another day interview, and the antibody against *Bartonella henselae* was positive at a later date, resulting in the definitive diagnosis of optic retinitis associated with CSD. The frequency of optic neuroretinitis in CSD is 1–2%, but according to Yap et al. [6], CSD is one of the most common infectious causes of infectious optic neuroretinitis.

CSD has a good prognosis with spontaneous recovery; therefore, it often requires no treatment. However, although the prognosis of visual acuity in neuroretinitis associated with CSD is generally considered good [1–3], cases with a long recovery time and fulminant visual impairment exist [4]. Thus, if the visual function is expected to be affected, aggressive treatment may be necessary. Some reports suggest that antimicrobial agents may alleviate symptoms and shorten the duration of the disease, and Reed et al. [2] suggest that such agents should be used as soon as the diagnosis is made. Although no established treatment exists, antibacterial agents, such as new quinolones, macrolides, and tetracyclines are often considered [1, 2, 4].

Furthermore, a certain type of immune response may be involved in the onset of the ocular lesions rather than direct infection with *Bartonella*; many studies have reported the concomitant use of corticosteroids in cases with ocular findings and strong visual dysfunction [3, 4]. However, there are no clear standards for dosage.

Although reports of pediatric cases exist [7–10], most infant cases were seen by ophthalmologists who noted subjective symptoms such as decreased visual acuity and visual field constriction. However, only a few cases of CSD have been reported in infants. Infants cannot complain of subjective symptoms (such as vision loss); therefore, these findings may be overlooked unless a fundus examination under mydriasis is performed. This may be because pediatricians do not usually consult an ophthalmologist, even in the presence of a systemic infection. In this case, the early detection of neuroretinitis by an ophthalmologist aided in the diagnosis of CSD. The change from cephem antibiotics to macrolide antibiotics, the addition of antituberculosis drugs, and the administration of steroids were effective in curbing the symptoms and restoring visual function.

Although the retinal and macular edema significantly improved, the macula may have atrophied or thinned due to the presence of hard exudates; thus, careful follow-up is required in the future. Further, photographing an infant's fundus is relatively challenging in daily medical care, and the record of the fundus findings greatly relies on the medical record description, which in turn, depends on the memory of the doctor who examined the patient. Fundus photography with a smartphone can be performed immediately without the need for expensive devices such as RetCam™ (Natus Medical Inc.), and it is also useful in observing changes over time.

## 4. Conclusion

In conclusion, the fever of unknown origin in this case was diagnosed based on a fundus examination. Moreover, since photographing an infant's fundus is extremely challenging, recording the fundus with a smartphone can be considered, which is relatively simple and useful for visualizing continuous changes.

## Data Availability

All data generated or analyzed during this study are included in this article. Further enquiries can be directed to the corresponding author.
